# LIVER TRANSPLANTATION FOR CARCINOMA HEPATOCELLULAR IN SÃO PAULO: 414 CASES BY THE MILAN/BRAZIL CRITERIA

**DOI:** 10.1590/0102-6720201600040007

**Published:** 2016

**Authors:** Gustavo Pilotto D. SÁ, Fernando P. P. VICENTINE, Alcides A. SALZEDAS-NETTO, Carla Adriana Loureiro de MATOS, Luiz R. ROMERO, Dario F. P. TEJADA, Paulo Celso Bosco MASSAROLLO, Gaspar J. LOPES-FILHO, Adriano M. GONZALEZ

**Affiliations:** 1 Postgraduation Program in Interdisciplinary Surgical Science, Federal University of São Paulo, São Paulo, SP, Brazil; 2; Sector of Liver Transplantation, Discipline of Surgical Gastroenterology, Federal University of São Paulo; São Paulo, SP, Brazil; 3 Sector of Liver Transplantation, Department of Surgery, Faculty of Medicine, University of São Paulo, São Paulo, SP, Brazil

**Keywords:** Liver transplantation, Hepatocellular carcinoma, MELD.

## Abstract

**Background:**

The criterion of Milan (CM) has been used as standard for indication of liver transplantation (LTx) for hepatocellular carcinoma (HCC) worldwide for nearly 20 years. Several centers have adopted criteria expanded in order to increase the number of patients eligible to liver transplantation, while maintaining good survival rates. In Brazil, since 2006, the criterion of Milan/Brazil (CMB), which disregards nodules <2 cm, is adopted, including patients with a higher number of small nodules.

**Aim:**

To evaluate the outcome of liver transplantation within the CMB.

**Methods:**

The medical records of patients with HCC undergoing liver transplantation in relation to recurrence and survival by comparing CM and CMB, were analyzed.

**Results:**

414 LTx for HCC, the survival at 1 and 5 years was 84.1 and 72.7%. Of these, 7% reached the CMB through downstaging, with survival at 1 and 5 years of 93.1 and 71.9%. The CMB patient group that exceeded the CM (8.6%) had a survival rate of 58.1% at five years. There was no statistical difference in survival between the groups CM, CMB and downstaging. Vascular invasion (p<0.001), higher nodule size (p=0.001) and number of nodules >2 cm (p=0.028) were associated with relapse. The age (p=0.001), female (p<0.001), real MELD (p<0.001), vascular invasion (p=0.045) and number of nodes >2 cm (p<0.014) were associated with worse survival.

**Conclusions:**

CMB increased by 8.6% indications of liver transplantation, and showed survival rates similar to CM.

## RESUMO

**Racional:**

O critério de Milão (CM) vem sendo utilizado como padrão para indicação do transplante hepático (TxH) por hepatocarcinoma (HCC) em todo mundo há quase 20 anos. Diversos centros têm adotado critérios expandidos com intuito de aumentar o número de pacientes candidatos ao transplante, mantendo bons índices de sobrevida. No Brasil, desde 2006, o critério de Milão/Brasil (CMB), que desconsidera nódulos <2 cm, é adotado, incluindo pacientes com maior número de nódulos pequenos.

**Objetivo:**

Avaliar o resultado do transplante hepático dentro do CMB.

**Métodos:**

Foram analisados os prontuários dos pacientes com HCC submetidos ao TxH em relação à recidiva e sobrevida através da comparação entre CM e CMB.

**Resultados:**

Em 414 TxH por HCC, a sobrevida em 1 e 5 anos foi de 84,1 e 72,7%. Destes, 7% atingiram o CMB através de downstaging, com sobrevida em 1 e 5 anos de 93,1 e 71,9%. O grupo de pacientes do CMB que excederam o CM (8,6%) teve sobrevida de 58,1% em cinco anos. Não houve diferença estatística na sobrevida entre os grupos CM, CMB e downstaging. A invasão vascular (p<0,001), tamanho do maior nódulo (p=0,001) e número de nódulos >2 cm (p=0,028) associaram-se com recidiva. A idade (p=0,001), sexo feminino (p<0,001), MELD real (p<0,001), invasão vascular (p=0,045) e o número de nódulos >2 cm (p<0,014) estiveram associados com a piora na sobrevida.

**Conclusões:**

O CMB aumentou em 8,6% as indicações de TxH e apresentou índices de sobrevida semelhantes ao CM.

## INTRODUCTION

The hepatocellular carcinoma (HCC) is the most common malignancy of the liver and the sixth leading cause of cancer mortality worldwide, with an incidence of 750,000 new cases per year. Liver transplantation (LTx) is currently considered the best treatment for the patient with liver cirrhosis and HCC^2^ ^,^ ^11^ ^,^ ^16^. 

The MELD criteria for liver transplant waiting list, adopted in Brazil since 2006, does not cover some serious liver diseases that lead to loss of liver function, such as hepatocellular carcinoma. These cases were classified as special situation and have received extra points, reaching the top of the waiting list for liver transplantation^5^ ^,^ ^12^ ^,^ ^15^ ^,^ ^23^.

With the special situation, patients with HCC began to take the place of patients with the most deteriorated and worse overall condition liver function. The challenge in adopting an extra score is seeking criteria that reduce the chance of being prioritizing patients with advanced HCC, with high risk of recurrence, instead of patients with higher MELD score ^6^ ^,^ ^22^ ^,^ ^24^ ^,^ ^29^.

In 1996, Mazzaferro et al. introduced the Milan criteria (MC). Cirrhotic patients with single nodule up to 5 cm or up to three nodules being the largest up to 3 cm, with no macrovascular invasion or detectable metastasis had a survival rate of 75% in four years^21^. The concepts identified in this study have been widely adopted and reproduced in transplant centers in the world, including Brazil, where the good results of survival in this population was reproduced^6^ ^,^ ^9^ ^,^ ^14^ ^,^ ^20^ ^,^ ^21^ ^,^ ^24^. However, only a small portion of patients with HCC fit into the MC, stimulating the development of expanded criteria, with satisfactory results^28^.

Thus, other broader criteria began to emerge (Table 1) in order to offer more patients with hepatocellular carcinoma the possibility of liver transplantation, expanding the limits determined by the MC^14^ ^,^ ^20^ ^,^ ^21^ ^,^ ^24^.

**TABLE 1:** Expanded criteria for liver transplantation for HCC

	n	Nodule	Size (cm)	Exams
Milan (16)	48	1 or 2 - 3	≤ 5 or ≤ 3 (each)	-
UCSF (19)	70	1 or 2 - 3	≤ 6,5 ou ≤ 4,5 (sum ≤ 8)	-
Navarro (20)	47	1 or 2 - 3	≤ 6 ou ≤ 5	-
Kyoto (21)	125	≤ 10	≤ 5 (each)	PIVKA-^II^< 400 (mU/mL)
Asan (22)	221	≤ 6	≤ 5 (each)	-
Edmonton (23)	52	-	Total volume ≤ 115 cm3	-
Valencia (24)	257	≤ 3	≤ 5 (sum ≤ 10)	-
Hangzhou (25)	195	-	Total ≤ 8 cm	-
			Total > 8cm	AFP ≤ 400, degree I/II (BX)
Up - to - seven (26)	1556	≤ 7	≤ 7 (soma)	-
Toronto (27)	294	Milan	Milan	-
		-	-	pathological criteria
Milan/Brazil	414	1 or 2 - 3	≤ 5 or ≤ 3 (each)	-
		(excluding nodules < 2cm)		

Some comparative studies have demonstrated the safety of expanded the Milan criteria, to include more patients without increasing the HCC recurrence rate^4^ ^,^ ^22^. The implantation of MC in Brazil was followed by minor modifications. Nodules < 2 cm are not considered, making this a modified MC adopted exclusively in Brazil, called Milan/Brazil Criteria (MBC).

The objective of this study was to evaluate the results of liver transplantation with special situation for HCC using the MBC, after the implementation of MELD criteria.

## METHOD

This study is registered in Brazil Platform and approved as the CEP 81706 of 21/08/2013.

Data were analyzed retrospectively by reviewing the medical records stored in the São Paulo State Transplantation Center of 414 patients undergoing LTx for HCC, conducted between January 2007 and December 2011 in the city of São Paulo.

The groups determined by preoperative imaging (downstaging, MBC and MC) were compared with each other and with respect to the results of survival and recurrence rates. The groups determined by pathology of explanted liver (MBC, CM and Out of Criteria) were compared with each other in relation to survival and recurrence rates.

### Statistical analysis

Data were analyzed descriptively. For categorical variables, absolute and relative frequencies were presented. The numerical variables were presented in summary measures (mean, quartiles, minimum, maximum and standard deviation). The tests included the Chi-Square, Student's t test, Mann-Whitney, Kruskal-Wallis, the Kappa coefficient and Kaplan-Meier survival curve. A p value <0.05 was considered statistically significant in all cases. Statistical analysis was carried out with SPSS 20.0 and STATA 12.

## RESULTS

There was a predominance of Caucasian patients (80%), males (79.5%) with mean age of 56 years. In January 2015, three hundred patients were alive (72.5%). If extra points were not used for HCC, the MELD would be 12.6 on average at the time of transplant. In this study, the patients were on the waiting list for LTx for a year on average.

Patients were submitted to different imaging in the preoperative period. Was selected for the analysis the last imaging test performed before transplantation. Computed tomography (58.7%) was the test of choice in most cases, followed by magnetic resonance imaging (31.9%) and ultrasonography (9.4%). These tests were, on average, made four months before the LTx.

All 414 study patients met the BMC based on preoperative imaging. Of these, 54 (7%) passed before by downstaging (Table 2). Of these, 33 patients (8.6%) would be out of the MC; however, there is a high correlation between the two criteria according to the Kappa coefficient (k=0.6 - p<0.001). 

**TABLE 2:** Distribution of patients by Milan criteria in accordance with Milan/Brazil criteria

Milan/Brazil criteria	Milan criteria			Total		
	No			Yes		
	n	%	n	%	n	%
Total	62	15,0%	352	85,0%	414	100,0%
No	29	100,0%	0	0,0%	29	100,0%
Yes	33	8,6%	352	91,4%	385	100,0%

Kappa 0,6 - p<0,00

The explant were sent to examination and analyzed the size and real number of nodes present in the explanted livers. Regarding pathology of explanted livers, 11.3% of patients were out of both criteria and 10.4% were within the MBC but exceeded the MC (Table 3). There was no statistically significant difference regarding the variables mortality and vascular invasion in patients included in the CMB, Milan criteria, or those submitted to downstaging.

**TABLE 3:** Distribution of patients by Milan criteria and Milan/Brazil criteria

Criteria	n	%
Criteria (imaging)	414	100,0
MBC	33	8,0
Downstaging	29	7,0
MC	352	85,0
MBC - (pathology)	414	100,0
No	47	11,3
Yes	367	88,7
MC - (pathology)	414	100,0
No	103	24,9
Yes	311	75,1

MBC=Milan/Brazil criteria; MC=Milan criteria

In Table 4, patients groups downstaging, Milan/Brazil Criteria and Milan Criteria, so classified by preoperative tests, were compared with the results of the pathology of explanted livers. Patients undergoing downstaging showed nodules larger than those previously classified by the Milan criteria. Patients classified as Criteria Milan/Brazil had higher total number of nodes compared to the other two groups.

**TABLE 4:** Comparison between the groups formed by preoperative imaging

Criteria (imaging)	Mean	SD	n	p	1° Quartil	Mediana	n	p
Size of the larger nodule* (pathology)				0,004				0,004
Downstaging	36,28^A^	18,82	29		23,00	35,00	29	
MC	27,77^B^	13,34	352		20,00	25,00	352	
MBC	24,72^B^	9,36	33		20,00	22,00	33	
Nodules >2 cm (pathology)				0,118				0,118
Downstaging	1,21	0,68	29		1,00	1,00	29	
MC	0,97	0,72	352		1,00	1,00	352	
MBC	1,21	1,17	33		0,50	1,00	33	
Nodule total number (pathology)				<0,001				<0,001
Downstaging	2,38	1,52	29		1,00	2,00	29	
MC	2,03^B^	1,52	352		1,00	1,00	352	
MBC	3,48^A^	2,50	33		2,00	3,00	33

p=descriptive level of the Kruskal-Wallis test; (A) and (B) show separate averages from Bonferroni-Dunn multiple comparisons; SD=standard deviation; *milimeters

To evaluate the effect of each of the numerical data variables, patient survival were adjusted to a simple Cox regression model (Table 5). There was a correlation between patient age (p=0.001), real MELD (p<0.001), number of nodules >2 cm in pathology (p=0.014) and survival time. Thus, every increase of one year in the age of the patient there is an increase of 4.4% in the risk of death and every 1-point increase in the real MELD, there is a 5% increase in the risk of death. Since every increase of 1 nodule >2 cm in pathology, there is an increase of 29.2% in the risk of death.

**TABLE 5:** Model results of simple Cox regression with patient data, imaging and pathology in relation to survival

	Risk ratio (IC95%)	p	n
Age	1,044 (1,019 - 1,069)	0,001	414
Real MELD	1,050 (1,023 - 1,078)	<0,001	414
Size of larger nodule (imaging)	1,003 (0,983 - 1,024)	0,744	379
Number of nodule >2 cm (imaging)	1,052 (0,657 - 1,687)	0,832	332
Number of nodule <2 cm (imaging)	0,944 (0,775 - 1,150)	0,567	413
Nodule total number (imaging)	1,019 (0,841 - 1,235)	0,848	331
Size of larger nodule (pathology)	1,006 (0,994 - 1,018)	0,326	413
Number of nodule >2 cm (pathology)	1,292 (1,052 - 1,586)	0,014	414
Number of nodule <2 cm (pathology)	1,035 (0,922 - 1,161)	0,562	413
Nodule total number (pathology)	1,086 (0,981 - 1,203)	0,113	413

**FIGURE 1:**
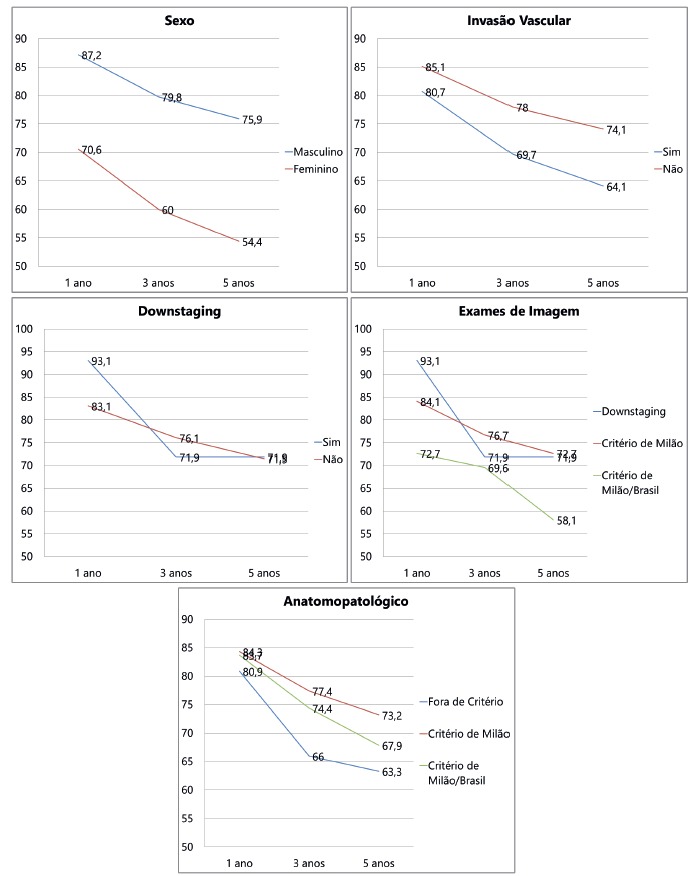
Results of the Kaplan-Meier survival analysis by patient characteristics, imaging and pathology

The survival analysis Kaplan-Meier showed significance in relation to gender. Male patients had higher survival than female (p<0.001). The association of increased survival in patients without vascular invasion identified by histopathologic study was demonstrated as well (p=0.045). The other analyzed factors have shown no difference in survival at 1, 3 and 5 years (Figure 1, Table 6).

**TABLE 6:** Kaplan-Meier survival analysis for patient data, imaging and pathology

	% Survival (years)			p
	1	3	5	
Gender				<0,001
Female	70,6 ± 4,9	60,0 ± 5,3	54,4 ± 5,7	
Male	87,2 ± 1,8	79,8 ± 2,2	75,9 ± 2,5	
Vascular Invasion				0,045
No	85,1 ± 2,0	78,0 ± 2,4	74,3 ± 2,7	
Yes	80,7 ± 3,8	69,7 ± 4,4	64,1 ± 4,7	
Downstaging				0,961
No	83,1 ± 1,9	76,1 ± 2,2	71,5 ± 2,4	
Yes	93,1 ± 4,7	71,9 ± 8,5	71,9 ± 8,5	
Criteria (imaging)				0,381
MBC	72,7 ± 7,8	69,6 ± 8,0	58,1 ± 10,1	
Downstaging	93,1 ± 4,7	71,9 ± 8,5	71,9 ± 8,5	
MC	84,1 ± 1,9	76,7 ± 2,3	72,7 ± 2,5	
Criteria (pathology)				0,279
Out of Criteria	80,9 ± 5,7	66,0 ± 6,9	63,3 ± 7,1	
MBC	83,7 ± 5,6	74,4 ± 6,7	67,9 ± 7,5	
MC	84,3 ± 2,0	77,4 ± 2,3	73,2 ± 2,6	

Estimate the probability of survival±SE; p=descriptive level of Log Rank test (Mantel-Cox)

During the study period, were observed (7.2%) thirty recurrence of HCC and there was an association only between recurrence and vascular invasion (p<0.001). Thus, it was noted a higher rate of recurrence in patients with vascular invasion (16.5%) compared to those without this condition (4.0%). For the other variables, there were no associations (Table 7).

**TABLE 7:** Distribution of patients for recurrence and Odds Ratio

	Recurrence				Total		p	Odds Ratio
	No		Yes					
	N	%	N	%	N	%		
Gender	384	92,8%	30	7,2%	414	100,0%	0,071	
Female	75	88,2%	10	11,8%	85	100,0%		2,06
Male	309	93,9%	20	6,1%	329	100,0%		1,00
Vascular Invasion	381	92,7%	30	7,3%	411	100,0%	<0,001	
No	290	96,0%	12	4,0%	302	100,0%		1,00
Yes	91	83,5%	18	16,5%	109	100,0%		4,78
Criteria (imaging)	384	92,8%	30	7,2%	414	100,0%	0,107^a^	
Downstaging	24	82,8%	5	17,2%	29	100,0%		2,98
MC	329	93,5%	23	6,5%	352	100,0%		1,00
MBC	31	93,9%	2	6,1%	33	100,0%		0,92
Criteria (pathology)	384	92,8%	30	7,2%	414	100,0%	0,139^a^	
Out of criteria	40	85,1%	7	14,9%	47	100,0%		2,53
MC	303	93,5%	21	6,5%	324	100,0%		1,00
MBC	41	95,3%	2	4,7%	43	100,0%		0,70

p=descriptive level of chi-square test or Fisher's exact test^a^

There were significant differences in the variables: larger nodule size (p=0.005), number of nodules <2 cm (p=0.026), number of nodes >2 cm (p=0.028) and size of the largest nodule (p=0.001) in relation to recurrence rate. Thus, on average, patients with recurrences had higher values in these variables than those without recurrence. Moreover, the average number of lower nodes 2 cm was lower than in-group of patients with recurrence in the patients without recurrence (Table 8).

**TABLE 8:** Summary of variable by recurrence occurrence

HCC Recurrence	Mean	SD	n	p
Age (years)	56,39	8,72	414	0,052
No	56,16	8,84	384	
Yes	59,37	6,40	30	
Real MELD	12,64	5,75	414	0,448
No	12,56	5,60	384	
Yes	13,63	7,47	30	
Number of nodule >2 cm (imaging)	1,13	0,40	332	0,487^a^
No	1,12	0,39	306	
Yes	1,19	0,49	26	
Size of larger nodule* (imaging)	27,91	9,39	379	0,005^a^
No	27,60	9,47	353	
Yes	32,04	7,19	26	
Number of nodule <2 cm (imaging)	0,63	0,97	413	0,026
No	0,65	0,99	383	
Yes	0,37	0,61	30	
Nodule, total number (imaging)	1,69	1,03	331	0,784^a^
No	1,69	1,05	305	
Yes	1,62	0,75	26	
Number of nodule >2 cm (pathology)	1,00	0,77	414	0,028
No	0,98	0,77	384	
Yes	1,30	0,70	30	
Size of larger nodule* (pathology)	28,13	13,71	413	0,001
No	27,53	13,22	383	
Yes	35,83	17,29	30	
Number of nodule <2 cm (pathology)	1,17	1,53	413	0,324
No	1,19	1,55	384	
Yes	0,90	1,32	29	
Nodule, total number (pathology)	2,17	1,66	413	0,992
No	2,17	1,69	384	
Yes	2,17	1,34	29	

*=measured in millimeters; p=descriptive level of the Student t test or Mann-Whitney

## DISCUSSION

Most patients were men (79.5%), white (80%), middle-aged (mean age 56 years), mildly overweight (BMI=26.5) and low value of MELD (average 12.7). Females in the sample showed a lower survival in Kaplan-Meier curve for 1, 3 and 5 years (p<0.001) when compared to males (Table 6). This result contradicts what is reported in the medical literature. Duffy et al. demonstrated relative risk of mortality of 2.43 for men (p<0.023); however, this variable was not significant in the multivariate analysis^24^. There is little data available in the literature relating gender as a prognostic factor, but few studies have reported female gender as a protective factor for recurrence and mortality, determining improved survival rate^8^ ^,^ ^18^ ^,^ ^19^.

The patient's age is presented as a factor associated with survival (p=0.001). For each increase of one year of age, there is an increase of 4.4% in the risk of death (Table 5). Other studies have identified age as a prognostic factor of mortality. Adler et al. in the European study for liver transplantation survival, in 226 patients, identified the age of 50 years as a risk factor for increased mortality^1^. 

A few studies have linked real MELD with survival of the transplanted patient, since this value has no influence on the waiting list due to the extra marks for HCC^12^ ^,^ ^26^. Todo et al. in Japan, presented a study in patients with HCC underwent living donor liver transplantation demonstrating that the real MELD influenced survival^26^. The results of this study validate the association of real MELD with survival time with p<0.001. For each 1-point increase in the real MELD, there is an increase of 5% in the risk of death (Table 5).

The waiting time for LTx for HCC patients is crucial for a better prognosis since the progression of the disease can exclude it from the queue for transplantation if nodules size or number exceed the MC. This rate ranges from 7-11% in 6 months and approaches 40% at one year^30^. 

Some authors, however, suggest that the delay in the waiting list can provide a better selection of candidates for LTx. Patients with aggressive tumor behavior, and increased risk of recurrence present a greater risk of drop out due to faster progression of neoplasia and would not be transplanted^25^. Recent studies have shown increased survival after liver transplantation for HCC in patients who waited longer in the awaiting list^14^ ^,^ ^25^. 

Apparently, patients with HCC have an advantage in the current organ allocation system, when compared with patients without tumor, raising the question of including biological factors of poor prognosis, such as alpha-fetoprotein, and tumor growth rate^4^. Currently, the proportion of transplantation for HCC in São Paulo is around 33% of total LTx. This scenario is worrying because it coincides with increased mortality in the awaiting list ^6,12,22,29^. 

This analysis shows no association of time on the waiting list and survival. The mean time for the liver transplantation was 11.5 months in patients alive and 13 months in patients who died (p>0.1).

Comparing the CM, CMB and downstaging groups, no difference was detected regarding the occurrence of vascular invasion (p= 0.501). In accordance with what is widely reported in the literature, vascular invasion showed association with decreased survival. There was a significant difference (p 0.045), with 5-year survival of 74.3% for the group without vascular invasion and 64.1% for the group with vascular invasion. Similarly, the HCC recurrence rate is significantly associated with the presence of vascular invasion detected on pathology (p<0.001).

Following the release of the MC by Mazzaferro et al. in 1996, transplantation in HCC began to show better results, since patients with HCC to respect the limits of one nodule ≤5 cm or even 3 cm nodules ≤3^21^. Most expanded criteria shows increasing the size and number of nodules in a controlled manner would result a recurrence risk and survival rate comparable to the MC^7^ ^,^ ^9^ ^,^ ^14^ ^,^ ^20^ ^,^ ^21^ ^,^ ^24^ ^,^ ^27^.

MBC disregards the counting of nodules smaller than 2 cm and thus offers the possibility of liver transplantation to patients with higher number of small nodules, which would exceed most of the criteria cited above. Table 3 shows the distribution of patients according to MBC, MC and downstaging.

Note that only a small proportion of patients selected for liver transplantation, the CMB has exceeded CM (8.6%), there is a strong correlation between the two criteria.

Analyzing the groups, the hypothesis that the MBC group has a higher number of nodules on biopsy compared to the MC is confirmed. This significant difference can be explained by the presence of nodules smaller than 2 cm, which are not accounted for at the time of indication for liver transplantation, but are identified on pathology of the explanted liver.

Applying the MBC and MB to results from the pathology of explanted livers, the formation of other three groups can be observed, adding to the first two groups the Out of Criteria group (OC, Table 3). This group corresponds to 47 patients (11.3% of the sample) who did not fulfill neither of the two allocation criteria and therefore configure the failure rate in the Brazilian allocation system. Other studies showed a failure rate that ranged from 18-40%^1^ ^,^ ^4^ ^,^ ^10^. 

There was no difference in patient survival between the MBC and MC groups. Patients transplanted in the MBC had a survival rate of 72.6% at five years, comparable to the most important services in the world^14^. Considering only those patients who exceeded the MC, the survival rate drops to 58.1%, but without statistical significance. The decrease in survival is probably associated with higher number of identified nodules in the MBC group. Several studies of risk factors for post-LTx mortality from HCC indicate to multiple nodules as a criterion of poor prognosis^2^ ^,^ ^16^ ^,^ ^10^. 

There was no statistical difference in vascular invasion and survival when compared both groups, submitted or not to downstaging, respectively, 71.9% and 71.5% survival at five years (Figure 1). This result confirms the data widely available in the literature^3^ ^,^ ^7^ ^,^ ^13^ ^,^ ^17^.

The recurrence was associated with the size of the largest nodule, both measured by imaging preoperative (p=0.005) as the pathology of the explanted liver (p<0.001). Patients with larger nodules showed a higher recurrence rate. The number of nodules larger than 2 cm identified in pathology also been associated with the recurrence of HCC after liver transplantation (p<0.028).

## CONCLUSION

The CMB increased by 8.6% indications of liver transplantation, and showed survival rates similar to CM.
